# Addition of L-arginine in skim milk extender maintains goat spermatozoa quality in chilled temperature for five days

**DOI:** 10.14202/vetworld.2019.1784-1789

**Published:** 2019-11-15

**Authors:** Suherni Susilowati, Indah Norma Triana, Wurlina Wurlina, Arimbi Arimbi, Pudji Srianto, Imam Mustofa

**Affiliations:** 1 Department of Veterinary Reproduction, Faculty of Veterinary Medicine, Universitas Airlangga, Kampus C Unair, Jl. Mulyorejo, Surabaya - 60115, Surabaya, Indonesia; 2 Department of Veterinary Pathology, Faculty of Veterinary Medicine, Universitas Airlangga, Kampus C Unair, Jl. Mulyorejo, Surabaya - 60115, Surabaya, Indonesia

**Keywords:** apoptosis, goat spermatozoa, L-arginine, malondialdehyde level, necrosis

## Abstract

**Aim:**

The purpose of this study was to determine the benefits of L-arginine addition in skim milk extender to maintain the quality of goat spermatozoa in chilled storage.

**Materials and Methods:**

A total of 18 ejaculates from three healthy goats with weight and age of 45 kg and 4-5 years, respectively, were divided into three groups. The control group contained goat semen diluted in a skim milk extender without L-arginine; Treatment I and Treatment II contained goat semen diluted in a skim milk extender with added L-arginine 4 and 6 mM, respectively. These three groups were chilled at 5°C and evaluated daily for 5 days. Observed variables were viability, motility, intact plasma membrane (IPM), malondialdehyde (MDA) level, necrosis, and apoptosis of spermatozoa.

**Results:**

The addition of L-arginine 4 mM was the best treatment in maintaining viability, motility, and IPM and a decreased MDA level, percentage of necrosis, and apoptosis of goat spermatozoa. An ejaculate in this extender can be divided into 37 doses for intracervical insemination in <1 ml volume with 125 million motile spermatozoa.

**Conclusion:**

Goat semen retained its quality when kept for 5 days in chilled storage by adding L-arginine in skim milk extender.

## Introduction

Etawa goat is a popular name in Indonesia for Jamnapari (or Jamunapari), a breed of goat originating from the Indian subcontinent. This breed is distributed almost all around the island of Java, Indonesia, for both milk and meat. The average age of first mating is 18 months, with conception rate at nearly 90% by natural mating, commonly resulting in triplet or quadruplet pregnancy. The average milk yield is almost 2 kg/day. With these advantages, Etawa goats are better bred by artificial insemination techniques than natural mating. Artificial insemination technology has been developed in Indonesia with excellent results, especially in cattle and dairy cows. However, the application of this technology in small ruminants is still minimal. In the future, artificial insemination will play an essential role in breeding programs of sheep or goats in Indonesia.

One contributing factor in the effort to optimize the program of artificial insemination in goats is the availability of frozen semen. However, the freezing process of goat spermatozoa causes more damage to membrane structure and function as well as the sperm’s ability to sustain life [1]. Alternatively, artificial insemination in goats can be conducted using chilled semen. Semen is cooled to 5°C and stored for several days until it is used for insemination [2,3]. In bull semen, preservation at 5°C for 22 h results in a higher quality of post-thawed sperm (motility, viability, and membrane integrity), and lower malondialdehyde (MDA) and DNA damage, compared to those of 4 h [4]. The problem is that the life span of the spermatozoa after ejaculation is only 8 h and is mediated by increasing concentration of lactic acid, a metabolic product of the spermatozoa itself, which causes lipid peroxidation [5,6]. L-arginine is an amino acid that plays a role as an antioxidant that produces nitric oxide (NO), thereby reducing sperm membrane lipid peroxidation.

Therefore, this study was conducted to determine the best dose of L-arginine addition in skim milk extender based on the viability, motility, intact plasma membrane (IPM), MDA level, necrosis, and apoptosis of goat sperm storage at 5°C in 5 days.

## Materials and Methods

### Ethical approval

This study is excerpted from the ethical clearance examination of the Animal Care and Use Committee because semen collection using artificial vagina does not affect the normal physiology of goat.

### Experimental animals

The study was conducted in the Teaching Farm of Faculty Veterinary Medicine, Universitas Airlangga, located at Gresik District, East Java, Indonesia. A total of three Etawa goats were used for semen collection; the average weights and ages of the goats were 45 kg and 3-4 years old, respectively. Semen was collected from the goats 2 weeks after adaptation, 6 times for each goat, using an artificial vagina equipped with scaled glass containers. Immediately after collection, semen was brought to a laboratory to be examined macroscopically (volume, color, odor, consistency, and pH) and microscopically (mass movement, individual movement, viability, concentration, and hypo-osmotic swelling [HOS] test).

### Skim milk extender

Ten grams of skim milk was prepared, added to 100 ml water, heated at 92-95°C, then cooled to 20-27°C. Added to this was 1000 IU/ml of penicillin and 1 mg/ml of streptomycin [7]. The ejaculate of each goat at every collection was divided into three parts of equal volume and diluted in extender for a final sperm concentration of 400 million sperm cells/ml. The control group (CG) consisted of extender (without L-arginine) + goat semen; Treatment I (T1) of extender + L-arginine (4 mM) + goat semen; and Treatment II (T2) of extender + L-arginine (6 mM) + goat semen. All three treatments were chilled at 5°C and evaluated for percentage of sperm viability, motility, intact of the plasma membrane, MDA level, necrosis, and apoptosis daily for 5 days.

### Examination of variable

#### Viability

Fresh semen was dropped on glass, eosin-negrosin was added, mixed homogeneously, and a smear preparation was made and dried over the flame quickly. The viability of the sperm was examined with a microscope 400×. The percentage of dead and living spermatozoa was calculated in three microscopic fields of view. The assessment of spermatozoa viability was as follows: head of live spermatozoa appeared transparent or clear, meanwhile died spermatozoa suffered damage on the plasma membrane, caused increased permeability, dyes will enter the cell and head of spermatozoa look reddish [8].

#### Motility

A total of 10 µl of semen suspension was added with 10 µl of physiological NaCl, homogenized and dripped on a glass object, then covered with a cover glass. Counted progressive (forward) moving of spermatozoa was conducted using a light microscope 400 times magnification [8]. Progressive motility of spermatozoa was determined in percentage.

### IPM

The HOS test was used to evaluate plasma membrane integrity. As much as 1 ml of hypoosmotic solution (7.35 g Na citrate 2H_2_O, 13.52 g of fructose dissolved in 1000 ml of aquades) was added with 0.1 ml homogenized solution of diluted semen and then incubated at 37°C for 30 min and observed with a microscope 400×. Spermatozoa with IPM are characterized by a coiled spermatozoa tail because the plasma membrane of spermatozoa still functions well in water absorption in hypotonic environments, while spermatozoa with damaged membranes are characterized by a straight tail [9].

### MDA

The measurement of MDA levels (in nmol/ml) was carried out using the thiobarbiturate acid test method [5].

### Necrosis

The necrotizing spermatozoa were characterized by pyknosis, karyorrhexis, and nucleus karyolysis, as identified using hematoxylin-eosin staining [10]. Necrosis sperm determined of 100 spermatozoa in five microscopic fields of view at 400× and presented in percentage.

### Apoptosis

Examination of spermatozoa apoptosis used acridine orange staining. The spermatozoa preparations were first fixed with absolute methanol and glacial acid for 15 min, stained with acridine orange, and then observed with a fluorescence microscope at 100×. Spermatozoa with apoptosis were yellow to reddish, while the non-apoptotic ones were green [11].

### Statistical analysis

Data were tabulated according to the observed variables. The result of viability, motility, IPM, MDA level, necrosis, and apoptotic sperm was subjected to ANOVA, and if there was a difference, it was followed with less significance difference test at 95% level of significance.

## Results

This study used fresh goat semen, which was first examined macroscopically and microscopically. The macroscopic examination included volume, color, odor, consistency, and degree of acidity or pH. The microscopic examination included mass movement, individual movement, concentration, and viability of the spermatozoa (Table-1).

**Table-1:** Macroscopic and microscopic characteristics of fresh goat semen.

Indicators	Characteristics
Color	White yellowish
Odor	Typical
Consistency	Thick
pH	7.00
Volume (ml)	2.5±0.35
Concentration (million/mm^3^)	3995×10^6^
Mass motility	+++
Individual motility (%)	Progressive (85±3.50)
Viability (%)	91±2.45
Intact plasma membrane (%)	82±1.55

### Viability, motility, and dose for intracervical insemination

The addition of L-arginine in the milk extender produces a similar pattern of sperm viability and motility (Table-2). Semen extenders with the addition of L-arginine (T1 and T2) showed a higher (p<0.05) percentage of sperm viability and sperm motility compared to CG, either on the 1^st^ day until the 5^th^ day; the highest was in T1.

**Table-2:** Effect of L-arginine addition in skim milk extender and chilled for 5 days evaluation on viability and motility (%) of goat spermatozoa.

Parameters	Day 1	Da y 2	Day 3	Day 4	Day 5
Viability					
CG	80.40±2.08^c^	68.17±4.49^c^	50.50±2.24^c^	41.50±3.24^c^	39.33±2.50^c^
T1	89.33±1.45^a^	79.83±4.23^a^	70.83±2.22^a^	58.51±3.08^a^	47.83±3.08^a^
T2	82.50±2.17^b^	76.50±2.50^b^	65.17±4.75^b^	52.33±3.23^b^	42.17±2.64^b^
Motility					
CG	70.50±3.08^c^	56.17±4.49^c^	45.50±5.24^c^	43.50±5.24^c^	38.23±3.50^c^
T1	87.33±3.45^a^	76.83±4.23^a^	69.83±7.22^a^	57.17±6.08^a^	46.53±3.08^a^
T2	80.50±2.17^b^	70.50±3.50^b^	62.17±4.75^b^	50.33±3.23^b^	41.17±2.64^b^

Values with different superscripts in the same column of viability and motility variable are significantly different (p<0.05). CG=Control Group, T1 and T2 diluted semen supplemented with L-arginine at 4 and 6 mM, respectively

Our field experience was using a dose of 125 million motile spermatozoa for intracervical insemination on goats and ewes. In this study, we could calculate the average of motile spermatozoa per ejaculation, i.e., the average of ejaculate volume (ml) multiply with spermatozoa concentration (million/ml) multiply with motility (%). The number of intracervical insemination dose counted was based on the number of motile sperm divided by 125 million. Meanwhile, the volume of one dose for intracervical insemination was the volume of extended semen divide with the number of doses (Table-3). The supplementation of 4 mM L-arginine in the skim milk extender (T1) had the highest motile spermatozoa, although, on the 5^th^ day of storage at 5°C, it could be divided into 37 doses with <1 ml volume for intracervical insemination.

**Table-3:** Effect of L-arginine addition in skim milk extender chilled for a 5-day evaluation on the number of motile goat spermatozoa.

Group	Day 1	Day 2	Day 3	Day 4	Day 5
CG	7041.19±307.62 (56/0.44)	5609.98±448.44 (45/0.56)	4544.31±523.35 (36/0.69)	4344.56±523.35 (35/0.72)	3818.22±349.56 (31/0.82)
T1	8722.08±344.57 (70/0.36)	7673.40±422.47 (61/0.41)	6974.27±721.10 (56/0.45)	5709.85±607.24 (46/0.55)	4647.18±307.62 (37/0.67)
T2	8039.94±216.73 (64/0.39)	7041.19±349.56 (56/0.44)	6209.23±474.41 (50/0.50)	5026.71±322.60 (40/0.62)	4111.85±263.67 (33/0.76)

The numbers in brackets are the average of the doses with 125 million motile sperm cells for intracervical insemination/volume in ml

### Intact of the plasma membrane (IPM) and MDA

The IPM and MDA level in this study is illustrated in Table-4. The additional 4 mM of L-arginine (T1) in semen extenders showed the highest percentage of the IPM, and lowest MDA level, either on days 1, 2, 3, 4, and 5 (p<0.05) compared to CG and T2.

**Table-4:** Effect of L-arginine addition in skim milk extender chilled for a 5-day evaluation on the intactness of the plasma membrane (IPM) and the MDA level (nmol/ml) of goat spermatozoa.

Parameters	Day 1	Day 2	Day 3	Day 4	Day 5
IPM					
CG	53.00±2.83^c^	45.33±2.16^c^	35.07±2.87^c^	20.83±2.79^c^	15.83±2.63^c^
T1	68.00±2.10^a^	56.53±2.31^a^	49.33±2.25^a^	35.67±2.50^a^	22.67±1.63^a^
T2	57.08±2.16^b^	50.50±3.27^ab^	41.50±2.33^b^	22.17±2.17^b^	18.83±3.72^b^
MDA					
CG	2305.25±52.83^c^	2510.35±32.16^c^	2805.17±42.87^c^	3490.17±22.79^c^	3876.17±42.25^c^
T1	1636.43±42.39^a^	1786.34±12.31^a^	2056.45±32.25^a^	2375.35±42.50^a^	2850.25±31.23^a^
T2	1845.34±32.17^b^	2095.45±33.27^b^	2563.24±32.10^b^	2945.35±52.20^b^	3245.25±23.45^b^

Values with different superscripts in the same column of IPM and MDA variables are significantly different (p<0.05). CG=Control group, T1 and T2 diluted semen supplemented with L-arginine at 4 and 6 mM, respectively, MDA=Malondialdehyde, IPM: Intact plasma membrane

### Necrosis and apoptosis

Diluted semen without the addition of L-arginine (CG) showed the highest percentage of necrosis and apoptosis of spermatozoa in all days of study, followed by T2, and the lowest was T1 (p<0.05) (Table-5).

**Table-5:** Effect of L-arginine addition in skim milk extender chilled for a 5-day evaluation on the necrosis and apoptosis (%) of goat spermatozoa.

Treatment	Day 1	Day 2	Day 3	Day 4	Day 5
Necrosis					
CG	18.83±3.82^c^	26.50±2.33^c^	29.05±1.86^c^	35.17±2.71^c^	39.17±2.76^c^
T1	5.67±3.50^a^	8.17±3.43^a^	15.50±1.14^a^	20.50±4.23^a^	23.33±2.41^a^
T2	16.50±1.76^b^	20.00±2.89^b^	22.83±2.87^b^	25.33±3.88^b^	29.50±3.15^b^
Apoptosis					
CG	17.83±3.62^c^	25.50±2.33^c^	29.05±1.76^c^	36.15±2.71^c^	39.27±2.76^c^
T1	7.67±3.50^a^	9.17±3.43^a^	15.50±1.14^a^	21.50±4.23^a^	24.33±2.21^a^
T2	12.50±1.76^b^	20.00±2.59^b^	22.83±2.37^b^	25.33±3.68^b^	29.50±3.15^b^

Values with different superscripts in the same column as the necrosis and apoptosis variables are significantly different (p<0.05). CG=Control group, T1 and T2 diluted semen supplemented with L-arginine at 4 and 6 mM, respectively

## Discussion

The fresh goat semen in this study has been qualified to be processed for artificial insemination use by diluting semen based on ejaculate volume and percentage of motility (Table-1). In goats with an ejaculate volume of more than 0.5 ml and motility of more than 70% can be used for artificial insemination with fresh or diluted semen. Decreased spermatozoa fertility will occur depending on the length of storage [12].

### Viability, motility, and dose for intracervical insemination

The high motility of spermatozoa requires high energy derived from mitochondrial metabolism, which results in the high level of reactive oxygen species (ROS) that harms spermatozoa viability. The small volume of sperm cytoplasm makes it challenging to transport antioxidant enzymes, such as glutathione peroxidase, catalase, and superoxide dismutase into other compartments in the spermatozoon to address ROS [13].

Spermatozoa metabolism increases with the presence of L-arginine [14]. L-arginine increases NO in cells that are synthesized by NO synthases, an isoenzyme family in mitochondria [15]. NO plays a role in the absorption of oxygen for cell metabolism and increases sperm motility [16], and helps induce acrosome reactions, sperm chemotaxis, and sperm-egg interaction [17,18]. Low doses of L-arginine induce glucose metabolism, high consumption of substrate, and finally impact higher sperm motility (T1 of Table-2) and prevent lipid peroxidation [19]. In contrast, high L-arginine concentration reduces sperm motility [16,17] and adversely affects the quality of spermatozoa in long-term storage, as shown in T2 of Table-2 [14].

The decrease in temperature and duration of storage causes a reduction in the viability and motility of spermatozoa mediated to biochemical changes by cold shock, osmotic disorders, and cell membrane damage due to oxidative stress [20]. In the process of mitochondrial oxidative phosphorylation, nicotinamide adenine dinucleotide is an electron donor, and oxygen is an electron acceptor in the electron transport chain. The process is an oxidation and reduction reaction in the synthesis of adenosine triphosphate [21], energy for sperm motility. The electrophilic aldehydes generated as a result of lipid peroxidation leads to a rapid loss of sperm motility [22].

The ROS plays a role in regulating sperm function and fertilization [23]. Physiologically, ROS is essential for regular purposes of spermatozoa, such as maturation, hyperactivation, acrosome reaction, and spermatozoa-oocyte fusion [24]. In viable and fertile sperm, both in fresh and diluted semen, ROS levels, and membrane lipid peroxidation are low [25]. At low levels, ROS plays a decisive role in tyrosine phosphorylation, sterol oxidation, and cholesterol efflux from the plasma membrane in the spermatozoon capacitation and the fertilization process [26].

A dose of 125 million motile spermatozoa was optimum for goat and ewes’ intracervical insemination [27,28]. In this study, the supplementation of 4 mM L-arginine in the skim milk extender (T1) had the most amount of motile spermatozoa, although, on the 5^th^ day of storage at 5°C, it was viable for 37 doses of intracervical insemination, with <1 ml volume per dose.

### IPM and malondialdehyde level

Intact, the plasma membrane (IPM) has a negative correlation to MDA and is positively correlated with the viability and motility of spermatozoa. The low dose of L-arginine results in higher viability and motility of spermatozoa; mediated, it increases the antioxidant capacity of semen, which results in a decrease in MDA [29].

The lipid of goat sperm’s plasma membrane has a higher concentration of unsaturated fatty acids compared to other ruminants. Therefore, in the chilling process, the lipid of the plasma membrane can be damaged, resulting in lipid peroxidation. Susceptibility to cold temperatures is associated with a higher ratio of unsaturated fatty acids than saturated fats, which results in the formation of a highly ROS [30]. One of the products of lipid peroxidation is MDA [26], which increases in long-term storage.

### Necrosis and apoptosis

Necrosis and apoptosis of spermatozoa seemed positive correlated with MDA level, and negatively correlated with viability, motility, and IPM. Necrosis is acute and irreversible cell death, caused by the inability of the cells to adapt. Due to the damage to the plasma membrane, it is unable to maintain the homeostasis that allows the entry of water and extracellular ions [31]. Cell necrosis may occur due to the activity of lysozyme, an enzyme product of lysosome. It digests the cell membranes such as the mitochondria membrane, ribosomes, and other cell apparatus, including intracytoplasmic fluid so that damage is caused to the cells, followed by cell lysis [32].

Apoptosis is a programmed cell death that is not preceded by cell swelling or inflammation. Apoptotic cells will shrink due to a breakup of the cell nucleus and the chromosomes, which all form apoptotic bodies. The apoptosis is triggered by lipid peroxidation, which leads to the activation of ROS in a continuous redox cycle. This cascade also includes the increased activity of a p53 protein, which will activate the Bax protein and then stimulate mitochondria to produce excessive cytochrome c that will cause apoptosis [32]. Cytochrome c is made up of apoptogenic proteins, such as the apoptosis-inducing factor and the endonuclease G [33], which cause a rapid decrease of sperm motility followed by caspase activation and exposure to phosphatidylserine on the surface of sperm a few hours later [34].

The nuclear DNA suffers oxidative damage during the chilling process, caused, among others, by the unique physical architecture of the spermatozoa and the limited cytoplasmic capacity that these cells possess for DNA repair. There was a correlation between the activation of caspase-3, an increased DNA fragmentation, and the lower fertilization rates of *in vitro* fertilization [35,36]. Damage of the DNA integrity of sperm during the chilling process is also related to the ROS [37].

The percentage of necrosis and apoptosis will be increased each day of storage, caused by the spermatozoa not being able to adapt to the changing composition of the extender. Simultaneously in the process of chilling, ROS increased, which affected the damage of DNA, especially the integrity of DNA as a cause of cell death [38]. The addition of L-arginine in low doses resulted in the lowest percentage of necrosis and apoptosis.

## Conclusion

The addition of 4 mM L-arginine in skim milk extender, which is stored in chilled temperature, maintains goat spermatozoa in the best quality (viability, motility, and IPM) as it inhibits elevation of MDA level, the percentage of necrosis and apoptosis of the spermatozoa. An ejaculate in this extender could be divided into 37 doses for intracervical insemination in <1 ml volume with 125 million motile spermatozoa.

## Authors’ Contributions

The SS compiled ideas and designed the main framework of this text as a part of research work under the supervision of IM and PS. SS and INT processed and evaluated the chilling of diluted goat semen. AA processed the measurement of MDA levels and staining for sperm necrosis and apoptosis. SS and WW conducted statistical analysis and conceived the manuscript. IM and PS critically read and revised the manuscript for intellectual content. All the authors read and approved the final manuscript.
